# How to estimate glomerular filtration rate in sub-Saharan Africa: design and methods of the African Research into Kidney Diseases (ARK) study

**DOI:** 10.1186/s12882-020-1688-0

**Published:** 2020-01-15

**Authors:** Robert Kalyesubula, June Fabian, Wisdom Nakanga, Robert Newton, Billy Ssebunnya, Josephine Prynn, Jaya George, Alisha N. Wade, Janet Seeley, Dorothea Nitsch, Christian Hansen, Moffat Nyirenda, Liam Smeeth, Saraladevi Naicker, Amelia C. Crampin, Laurie A. Tomlinson

**Affiliations:** 1 Medical Research Council/ UVRI & London School of Hygiene and Tropical Medicine Research Unit, Entebbe, Uganda; 2 Makerere University College of Health Sciences, Kampala, Uganda; 3 London School of Hygiene & Tropical Medicine, London, UK; 4 Wits Donald Gordon Medical Centre, Parktown, Johannesburg, South Africa; 5 Medical Research Council/Wits University Rural Public Health and Health Transitions Research Unit (Agincourt), School of Public Health, Faculty of Health Sciences, University of the Witwatersrand, Johannesburg, South Africa; 6 Malawi Epidemiology and Intervention Research Unit, Lilongwe, Malawi; 7 Department of Chemical Pathology, National Health Laboratory Services, University of Witwatersrand, Johannesburg, South Africa; 8 Department of Internal Medicine, Faculty of Health Sciences, University of Witwatersrand, Johannesburg, South Africa

**Keywords:** Measurement, Glomerular filtration rate, Chronic kidney disease, Sub-Saharan Africa

## Abstract

**Background:**

Chronic kidney disease (CKD) is a substantial cause of morbidity and mortality worldwide with disproportionate effects in sub-Saharan Africa (SSA). The optimal methods to estimate glomerular filtration rate (GFR) and therefore to determine the presence of CKD in SSA are uncertain. We plan to measure iohexol excretion to accurately determine GFR in Malawi, South Africa and Uganda. We will then assess the performance of existing equations to estimate GFR and determine whether a modified equation can better improve estimation of GFR in sub-Saharan Africa.

**Methods:**

The African Research on Kidney Disease (ARK) study is a three-country study embedded within existing cohorts. We seek to enrol 3000 adults > 18 years based on baseline serum creatinine. Study procedures include questionnaires on socio-demographics and established risk factors for kidney disease along with anthropometry, body composition, blood pressure, blood chemistry and urine microscopy and albuminuria. We will measure GFR (mGFR) by plasma clearance of iohexol at 120, 180 and 240 min. We will compare eGFR determined by established equations with mGFR using Bland-Altman plots. We will use regression methods to estimate GFR and compare the newly derived model with existing equations.

**Discussion:**

Through the ARK study, we aim to establish the optimal approach to estimate GFR in SSA. The study has the advantage of drawing participants from three countries, which will increase the applicability of the findings across the region. It is also embedded within established cohorts that have longitudinal information and serial measures that can be used to characterize kidney disease over a period of time. This will help to overcome the limitations of previous research, including small numbers, selected population sub-groups, and lack of data on proteinuria.

The ARK collaboration provides an opportunity for close working partnerships across different centres, using standardized protocols and measurements, and shared bio-repositories. We plan to build on the collaboration for this study for future work on kidney disease in sub-Saharan Africa, and welcome additional partners from across the continent.

## Background

Chronic kidney disease (CKD) is a substantial cause of morbidity and mortality worldwide with an estimated prevalence of 8–16% [1]. A recent systematic review estimated the prevalence of CKD in sub-Saharan Africa to be around 14% [2]. However, the optimal equation to accurately estimate glomerular filtration rate (GFR) among sub-Saharan African populations is uncertain. This uncertainty is a major barrier to identifying individuals with CKD and to estimation of disease burden.

Worldwide, several equations have been used to estimate GFR from serum creatinine [3–7]. Unfortunately, most of these have been derived from high-income countries with minimal validation in sub-Saharan African populations. Their accuracy at an individual level is limited, particularly in relation to the adjustments for African-American ethnicity, which might be related to variations in muscle mass. Serum creatinine levels at a given level of renal function vary substantially with ethnicity, age, sex, physical activity and nutritional status [8–14]. Several sources of inaccuracy in estimating GFR have been described, including biological variability in serum creatinine, laboratory induced errors in creatinine measurement and choice of estimating equation [15, 16]. The imprecision of creatinine measurement is more marked at values near the normal range where it is most critical to determine earlier stages of CKD [17–19]. Several methods for direct measurement of GFR are available through measurement of clearance of inulin, iothalamate, iohexol, and radio-active agents such as technetium-99 m diethylenetriamine penta-acetic acid (DTPA) and chromium-51 ethylene diamine tetra-acetic acid (EDTA), and 24-h urinary creatinine collection for estimation of creatinine clearance [20]. However, all of these have their challenges. DTPA, EDTA and inulin are expensive and not readily available in most African countries, while 24-h urinary collection is often inaccurate due to difficulty in ensuring a complete sample, coupled with the additional limitation of tubular creatinine secretion [20–22].

Iohexol is a readily available compound, which can be used to measure the GFR. Its advantages include low cost, excellent safety profile, low protein binding, low levels of toxicity at the dose used for measuring GFR, stability at room temperature (20 to 25 °C), and being able to provide an accurate measure of glomerular filtration [20, 23]. Previous studies using iohexol to measure GFR in sub-Saharan Africa have included few people with CKD [13, 24, 25]. The largest study to date conducted in the Democratic Republic of Congo and Ivory Coast used plasma iohexol to measure GFR in 494 participants. They noted that the African-American race coefficient did not improve the performance of creatinine-based GFR estimates of iohexol GFR. In particular, the Chronic Kidney Disease Epidemiology (CKD-EPI) and Modification of Diet in Renal Disease (MDRD) equations performed better without the race coefficient in participants with GFR ≥60 mL/min/1.73m^2^. The authors also evaluated the Full Age Spectrum (FAS) equation and found it to be as accurate as CKD-EPI for GFR ≥60 mL/min/1.73m^2^ but better for those with creatinine based GFR < 60 mL/min/1.73m^2^. Addition of cystatin C did not improve performance of the equations among this study group [25].

In the proposed study, we will use iohexol excretion in a population sample of 3000 participants with and without CKD to measure GFR and determine the optimal equation to estimate GFR in Uganda, Malawi and South Africa.

## Methods

### Primary objectives

1. To measure GFR using plasma clearance of iohexol
2. To assess the performance of existing eGFR equations, namely Cockcroft -Gault (CG), Modification of Diet in Renal Disease (4-v MDRD) and Chronic Kidney Disease-Epidemiology collaboration (CKD-EPI) by comparing these to measured GFR, among sub-Saharan Africans.

### Secondary objectives

In a Ugandan sub-sample, to assess the feasibility of using dry blood spot collection of iohexol in the determination of GFR.

In a Ugandan sub-sample to explore the current community understanding of kidney disease and available treatments from traditional healers and herbalists.

### Study organization and sites

**Fig. 1:**
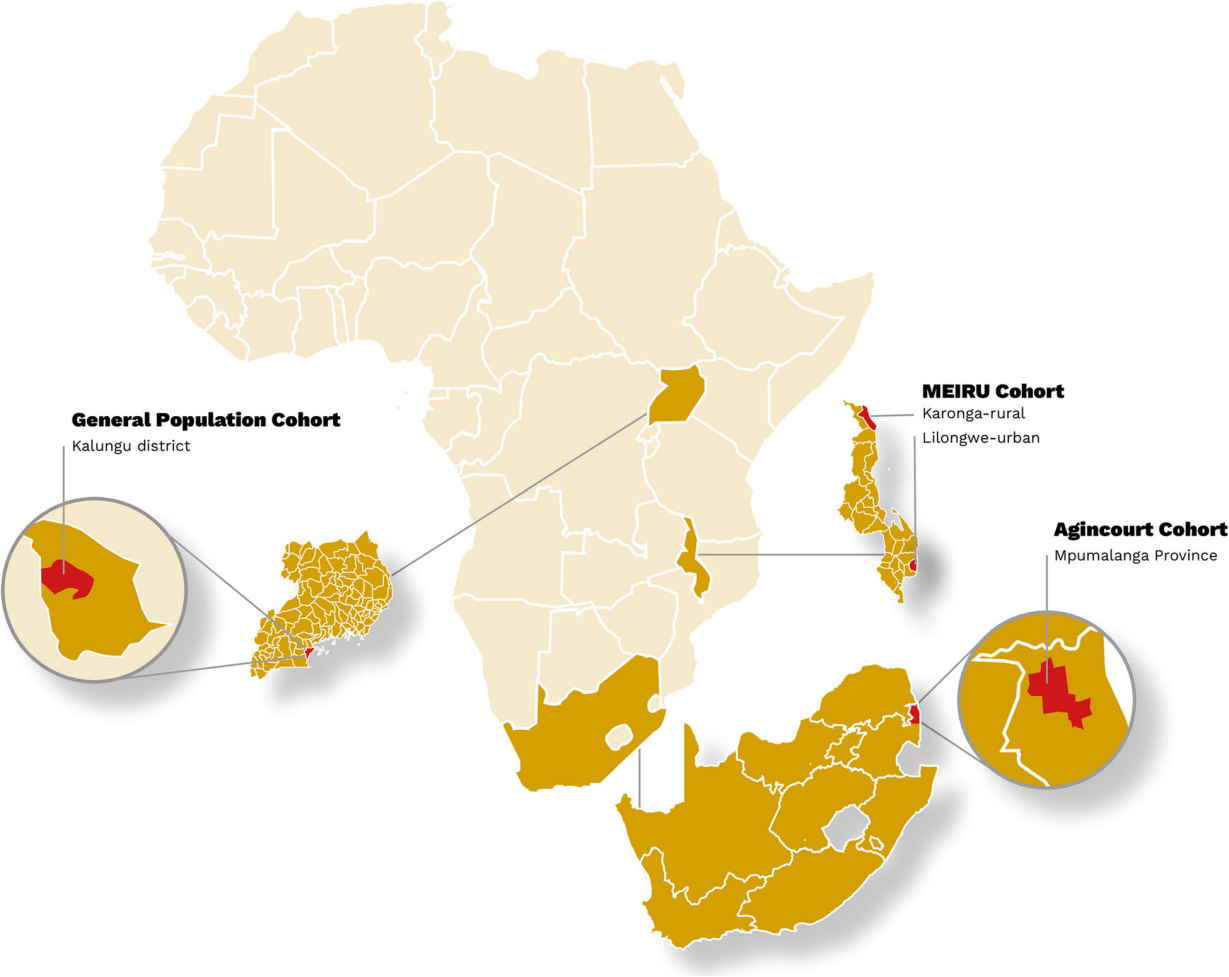
Map of Africa showing the ARK centres from Malawi, South Africa and Uganda. The general population cohort is run by the Medical Research Council-Uganda virus Institute and the London School of Hygiene and tropical Medicine (MRC-UVRI-LSHTM, Uganda). The Malawi Epidemiology and Intervention Research Unit (MEIRU) runs the MEIRU cohort in Malawi and the Medical Research Council/Wits University Rural Public Health and Health Transitions Research Unit runs the Agincourt Unit in South Africa. Map proposed by Robert Kalyesubula and illustrated by Helmut Kraus

We will have three participant countries across SSA (Fig. 1) and these centres have formed a new collaborative group, African Research on Kidney Disease (ARK) around this initial study.

A working committee will review the protocols for the design, conduct, progress and data collection and analysis plan. The committee will meet in person on several occasions to review protocols and measurement tools and to agree on a minimum dataset. The three centres have unique characteristics that will enrich the study with diversity among participants. The details of each of the different cohorts in which the current study will be nested are described below:

### Uganda

In Uganda, the study will be based in the General Population Cohort (GPC), originally established in 1989 [26]. The study area is located in rural south-western Uganda in one sub-county of Kalungu district, approximately 120 km from Entebbe town. The cohort comprises all residents of 25 adjacent villages, approximately 22,000 people with men and women in equal proportions, and 52% older than 13 years. The study population for the general population cohort is recruited through annual house-to-house rounds of the census where study participants are selected. Medical care is available through an established general clinic.

### Malawi

The Malawi Epidemiology and Intervention Research Unit (MEIRU) conducts ongoing epidemiological studies based at two sites, which will be used for recruiting participants for this study. The first is Karonga District in rural northern Malawi, predominantly subsistence farmers, fishermen and informal traders. The second site is Area 25 of Lilongwe, the capital city of Malawi. The urban area is socio-economically mixed and includes government and industry employees, traders and those in casual employment. We will enrol participants from both sites, providing a true population-wide sample and enabling urban: rural comparisons.

The stratified study sample was drawn from a pool of over 5000 individuals who were tested for serum creatinine as part of a larger survey (*n* = 33,000) of chronic conditions. Medical care is available through an established chronic care Non-Communicable Disease (NCD) clinic.

### South Africa

In South Africa, the study will be located within the Medical Research Council/University of the Witwatersrand Rural Public Health and Health Transitions Research Unit in Agincourt, Bushbuckridge sub-district of Mpumalanga province. The area includes a high-functioning health and socio-demographic surveillance system (HDSS) which covers approximately 115,000 people based on an annual update of resident status and vital events. The study setting also serves as a national pilot site for the development of integrated chronic disease care. The population still has gaps in access to electricity, water and tarred roads and unemployment rates are high, leading to high rates of labour migration. Patients will be managed through the primary health care system consisting of six clinics, two health centres and three district hospitals. The HDSS has a central clinic with advanced laboratory capacity, which will be used for this study.

### Study design

This will consist of cross-sectional studies embedded within ongoing cohorts in Malawi and Uganda and a longitudinal study in South Africa, all with serial bio-banking.

### Data collection, sample size and selection of sample

We intend to recruit 3000 participants in total. We have based the power calculation is based on the number of study participants needed to examine the accuracy of GFR estimating equations for ‘true’ GFR, at each of the three study sites separately. We have specified this as having 90% power to detect whether eGFR is within 5% of the iohexol value at an eGFR of 60mls/min, assuming a standard deviation of 25mls/min [9] and with a two-sided alpha of 0.05. This gives an estimated required sample size of 730 participants. Allowing for participants who do not wish to participate in this element of the study, and technical failures, we intend to recruit 1000 participants in each country.

In order to fulfil the aims of the study, a structured approach is needed. This will differ between South Africa and the other two sites (Malawi and Uganda).

### Details of site recruitment

We plan to recruit from each of the sites guided by the previous recruitment protocols. Each of the sites will however, have two phases of recruitment.

For the Uganda GPC, the first phase will consist of measurement of creatinine from stored sera.

Participant selection for this part of the study has been described elsewhere [27]. Briefly, participants were selected from the general population cohort, which is a community-based open cohort study of residents of 25 neighbouring villages. The participants are selected through house-to-house mobilisation and community surveys conducted through village-based hubs. For Uganda, 5979 individuals were identified for baseline creatinine measurement, which included all the adults surveyed in that round. We will use creatinine levels from the stored samples to stratify participants for the second phase of the study. Here, we will select participants according to eGFR cut offs of normal (>90mls/min/1.73m^2^); impaired renal function (60-90mls/min/1.73m^2)^ and low GFR (<60mls/min/1.73m^2^), all additionally stratified by age and sex. Selected participants will be approached by the community engagement lead (community mobilizer) for each village. Once participants assemble, the study team will take time to explain the details of the study to them, after which individual informed consent will be obtained. Participants will undertake a questionnaire to collect demographic data as well as history of known risk factors for kidney disease. Participants will also undergo detailed biophysical measurements and blood draws as detailed in the next sections. We will give participants an appointment to attend the medical clinic at the central Research Station, where iohexol measurements and other study tests will be performed.

For Malawi, for the first phase of the study, we will use creatinine assays from a previous survey to guide the selection of participants for the second phase of the study using a similar approach to that of Uganda. However, we will recruit participants 18 years and above from Malawi through household visits where similar procedures to those outlined above will be undertaken. We will carry out iohexol testing and other physical tests at a research clinic (phase 2). Details of participant recruitment have been detailed elsewhere [28].

**Fig. 2:**
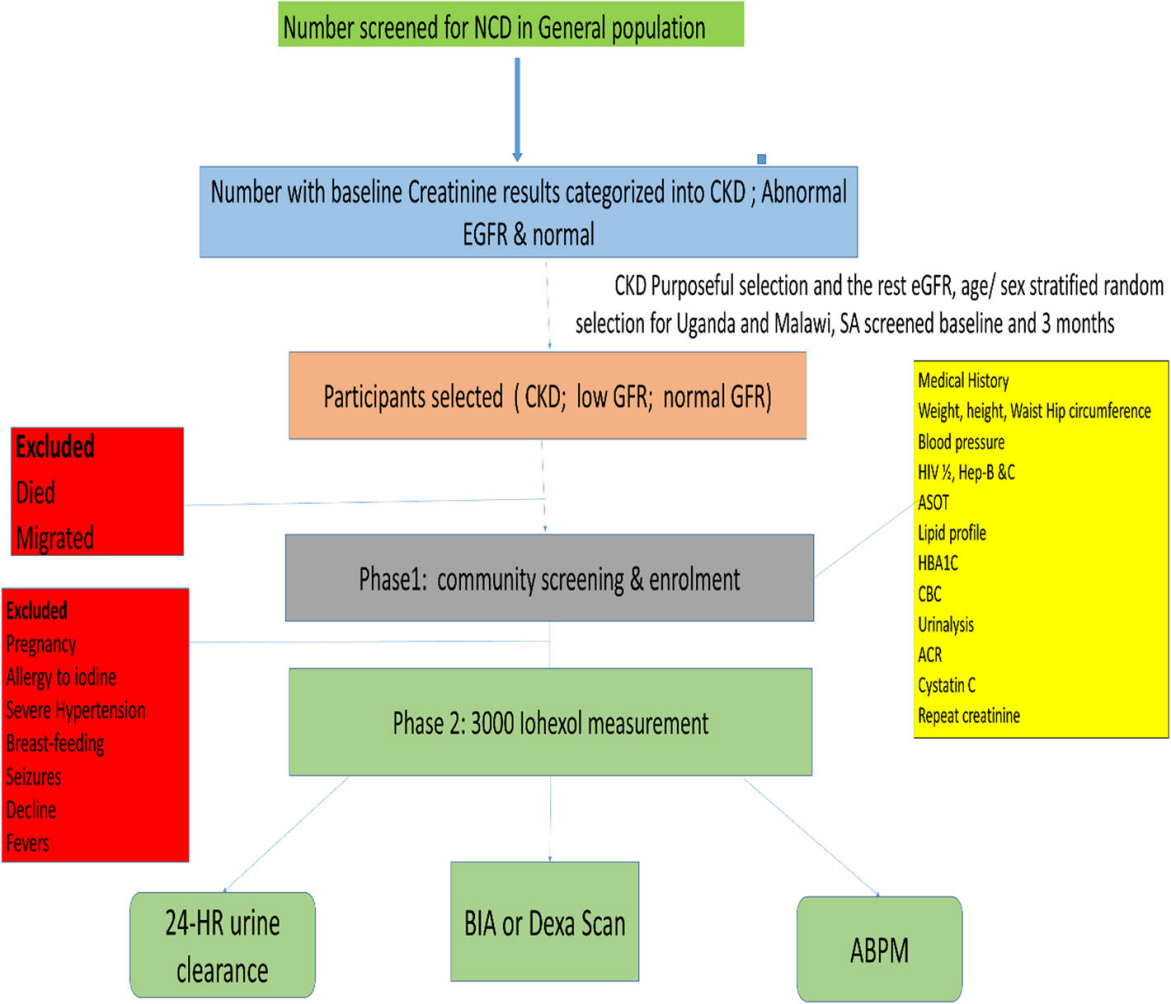
Recruitment flowchart for ARK. ABPM- Ambulatory Blood Pressure Monitor; ACR- Albumin: creatinine Ratio; ASOT- Antistreptococcal antibody titres; BIA- Bioimpedance Analysis; BP-Blood Pressure; CBC- Cell blood count; GFR- glomerular filtration rate; CKD- Chronic kidney disease; HB-Haemoglobin; NCD-Non-communicable diseases; SA-South Africa. Credit for the ARK study map and copyright go to Helmut Kraus

In South Africa, we will determine baseline creatinine and the prevalence of CKD using an age and gender stratified random sample of 2250 members of the rural Agincourt HDSS. The number of participants sampled in each stratum will be determined in order to ensure proportional allocation, based on the population demographic distribution. We will visit participants in their homes for the first phase of the study where the questionnaires, biophysical measurements and take blood draws to determine Human immunodeficiency virus (HIV) status, blood sugar level, lipid profile as well as the baseline creatinine level. We will repeat creatinine and urine albumin measures after a minimum period of 3 months to confirm CKD. For the second phase of the study, we will recruit participants from Phase 1 through household visits and give them a referral date to the clinic for iohexol measurement along with other tests (See Table 1 and Fig. 2 for details).

**Table 1:** Clinical and Laboratory measurements within the ARK study, by country

Type of information	Equipment	Field procedures	Participant type	Definition of variable	Site participation		
					Malawi	S. Africa	Uganda
Demographics	Questionnaire	Face-to-face interview	all	Sex, age, education status, marital status	x	x	x
Chronic disease history and family history	Questionnaire /medical records	Face-to-face interview	all	HTN, DM, CKD, Stroke, heart disease	x	x	x
Treatment history	Patient report/records	Face-to-face interview	all	HTN, DM, CKD, Stroke, HIV,TB, Heart disease, Cancer, Backache	x	x	x
Health behaviours	Patient report	Face-to-face interview	all	Tobacco use, alcohol consumption, Physical activity, vegetable, fruits, salt and water intake	x		x
Traditional medicine use	Questionnaire	Face-to-face interview	all	Use of herbs and traditional medicines	x	x	x
Physical examination	Omron® SECA® Flexible tape SECA© scales	Clinical Examination in the field, machines calibrated weekly	all	BP (mmHg), waist and hip circumferences (cms), height (m), weight (Kgs)	x	x	x
ABPM	ABPM spacelabs®	24 h BP, 30 min interval day, 1 h interval night	Sample per eGFR quarter	BP wake periods and sleep periods	x		x
BIA	Bodystat®	Tested in the field or clinic with participant, calibration weekly	all	Fat mass (kg), Lean mass (kg), Dry lean mass(kg), Total body water (L), Impedance at 50 KHZ (Ω)	x	x	x
DXA SCAN	Hologic Discovery A. QDR 4500 Series	Whole body scan performed in clinical research clinic during iohexol testing	all	Lean Mass (g), Fat mass (g) Fat % and BMI		x	
Ultra sound scan	Logiq e	Performed at clinic	all	Probe 4c for kidneys and bladder and probe12L CIMT probe		x	
ECG	ECG 300A®/ MAC600®	ECG protocol followed	all	LVH using the Sokolow-Lyon criteria	x	x	x
CBC	BC®	Venous blood draw	all	Total cell count, HB, MCV	x		x
24 HR URINE	Cobas Roche®	urine using small bucket.	Select patients for 24 h proteinuria, salt and feasibility.	Volume (mls) Protein (mg) Creatinine (mmol/L) Na^+^ mmol/L			x
Malaria screen	Malaria RDT®	Done in the community	all	Malaria infection	x		x
CRP	Cobas® & BC®	Blood draw	all	CRP	x		x
Hepatitis B	Cobas® ABBOTT (i1000SR)	Blood draw	all	HepB SAg	x	x	x
Hepatitis C	Cobas® (Uganda), HCV antibody rapid test (Malawi)	Blood draw	all	HepC Ab	x		x
ASOT	Cobas® & BC®	Blood draw	All	> 300			x
HIV screen	Alere Dertemine-Stat-pak-Bio line & Abbott Determine	MOH serial testing algorithm	all	HIV status	x	x	x
Schistosomiasis	Microscopy	Examination of centrifuged urine	all	Schistosoma hematobium	x	x	x
Microalbuminuria	CLINITEK® + analyser & Cobas® & BC®	ACR	all	Urine ACR	x	x	x
Urine analysis	Clinitek® & UroColor	Early morning urine	all	Protein, blood, glucose, WBCs, QuickVue Hcg Urine for pregnancy	x	x	x
Lipid profile	Cobas® & BC®	Blood draw	all	Cholesterol, LDL, HDL, TGs	x		x
RBS	BC®	Point of care testing (Uganda $ SA)	all	Diabetes > 11.1 or on medical treatment	x	x	x
HBA1C	Cobas® & BC®	Blood draw	All	6.5% or on treatment	x		x
Creatinine	Cobas® & BC®	Jaffe and IDMS	all	For eGFR estimation	x	x	x
Cystatin-C	Cobas®		all	For eGFR estimation	x	x	x
Iohexol	Omnipaque 300 mg I/ml Healthcare® HPLC®	Clinic based blood draws at 5, 120, 180 and 240 min after administration	all	Measured GFR	x	x	x
Iohexol, DBS	DBS®	DBS by finger prick at 120 and 240 min	300 selected by eGFR	Validation of measured GFR technique			x
Aldosterone/Renin	Cobas Roche®		Selected population		x		x

All biochemistry was tested using Cobas equipment in Uganda and South Africa and Beckman Coulter equipment in Malawi

*Abbreviations*: *ABPM* Ambulatory Blood Pressure Monitor, *ACR* Albumin Creatinine Ratio, *ASOT* Antistreptococcal antibody titres, *BC®* Beckman Coulter®, *BIA* Bioimpedance Analysis, *BP* Blood Pressure, *CBC* Cell blood count, *CKD* Chronic Kidney Disease, *CRP* C-Reactive protein, *CIMT* Carotid intima-medial thickness, *DBS* Dry blood sample, *DM* Diabetes Mellitus, *ECG* Electrocardiography, *eGFR* Estimated glomerular filtration rate, *HB* Haemoglobin, *HTN* Hypertension, *HPLC* High liquid pressure chromatography, *IDMS* Isotope dilution mass spectrometry, *LVH* Left ventricular hypertrophy, *MCV* Mean corpuscular volume, *MOH* Ministry of Health, *RBS* Random blood sugar, *SA* South Africa, *TB* Tuberculosis

### Inclusion criteria

- Adults aged 18 years and above from the three population cohorts
- Able to give informed consent

### Exclusion criteria

- Blood pressure > 180/110mmhg
- Pregnancy
- Breast-feeding mothers
- Known allergy to iodine−containing substances
- Uncontrolled seizures (defined as a seizure within the last 12 months).
- Acute febrile illness

### Demographic factors

We will collect data on demographic factors including age, sex, place of residence, education, occupation and livelihood, tobacco use, alcohol use, dietary history, physical activity. We will also take a medical history and treatment for chronic diseases including HIV, diabetes mellitus, hypertension, heart disease and CKD as well as previous and current use of traditional medicine and drugs.

### Physical examination

We will measure height, weight, waist circumference and hip circumference and calculate the body mass index (BMI) and the waist hip ratio accordingly. We will classify BMI according to WHO categories (weight/height^2^: kg/m^2^): underweight (< 18.5 kg/m^2^), normal weight (18.5–24.99 kg/m^2^), overweight (25.0–29.99 kg/m^2^) and obese (> 30.0 kg/m^2^).

We will undertake cardiovascular assessment through blood pressure and 24-h ambulatory blood pressure (BP) measurements (ABPM) on a sub-sample of participants (Malawi and Uganda), and electrocardiography (ECG). We will measure BP using Omron® M6 (for small, medium and large participants) and Omron HBP 110 machines (for obese participants). We will measure BP in triplicate after at least 5 min of rest and take the mean of the last two readings as the true blood pressure. We will derive BP classification from the National Institute of Health guidelines: where participants with a systolic BP greater than 120 mmHg but less than 140 mmHg, and/or a diastolic BP greater than 80 mmHg but less than 90 mmHg will be classified as pre-hypertensive. We defined hypertension as having a diastolic BP greater than or equal to 90 mmHg, systolic BP greater than or equal to 140 mmHg or being on treatment for high blood pressure. 24-h ambulatory blood pressure will be undertaken on a selected number of participants with no hypertension, pre-hypertension and hypertension across the spectrum of eGFR ranges and will capture wake and sleep periods. We will use the ECG for assessment of LVH using the Sokolow-Lyon criteria [29].

We will perform bioimpedance analysis (BIA) using the Bodystat® machine to measure body fat in relation to lean body mass for parameters outlined in Table 1. We will also perform a Dual-energy X-ray absorptiometry (DXA) scan for body composition in South Africa and use it to examine validity of the BIA measurements across countries.

### Laboratory investigations

The key laboratory measurement for this study will be iohexol (Omnipaque 300 and 350 mg I/mL, GE Healthcare) clearance. The iohexol measured GFR will serve as the gold standard for comparison with other methods.

Using aseptic technique study nurses will insert an IV line into the non-dominant arm of the participant and use the line for subsequent blood draws. We will administer a single dose of 5 ml of iohexol over 30 s through an intravenous cannula inserted in the dominant arm contralateral to the established IV line, followed by a 10 ml normal saline flush. We will weigh the iohexol dose in grams to a specificity of 0.01 g by weighing the syringe before and after administration of the iohexol. The research nurses will draw four milliliters of blood for the iohexol assay again at approximately 5, 120, 180 and 240 min after injection of iohexol, and record the exact time. We will calculate the GFR using a single compartment model based on iohexol clearance between 120 and 240 min.

In Uganda at the time points of 120 and 240 min, we will also collect a capillary sample of dried blood spots to compare to intravenous sampling of iohexol GFR as a modification from previous studies [11, 30]. Should this method prove to be accurate, it will greatly facilitate further studies in resource-limited settings. In order to avoid inter-laboratory variations, all plasma and blood spot samples of iohexol will be measured at the National Health Laboratory, Johannesburg, South Africa by ultra-pressure liquid chromatography-tandem mass spectrometry with identification and quantitation based on MRM transitions. The laboratory is accredited for the International Organization for Standardization (ISO) standard 15,189 and also participates in the internal quality control run at a high and a low level every 20 samples. The laboratory has participated in Equalis external control since 2017.

We will measure creatinine using the enzymatic method in Uganda and Jaffe for South Africa and Malawi standardized against an isotope dilution mass spectrometry (IDMS) method across all sites. We will adopt the recommended estimation and reporting format or measured renal function proposed by Fabian et al [31]. We will measure serum cystatin C to determine the accuracy of the cystatin-based CKD-EPI equations. We will measure Cystatin C using the standardized Roche Gen2 assay, which has been standardized against certified reference material (ERM-DA471/IFCC). To minimise batch variation, samples will be analysed at the end of the study at one central laboratory at MRC/UCRI&LSHTM for Uganda and Malawi while the samples in South Africa will be analysed at the National Health Laboratory, Johannesburg, South Africa. To understand laboratory differences and enable calibration we will cross-validate and measure 50-paired samples for creatinine in Uganda, Malawi and South Africa, and cystatin between South Africa and Uganda. Additional tests include full blood count (including haemoglobin level), tests for screening of infections (malaria, streptococcal infections, Hepatitis B, Hepatitis C and HIV Infection), C-reactive protein, random blood glucose, HbA1c and the fasting lipid profile as markers of metabolic risk.

We will perform four urine tests. We will take an early morning spot urine sample for quantitative urine albumin: creatinine ratio, an early morning urine dipstick analysis to qualitatively assess haematuria, leucocyturia and proteinuria, and microscopy will be performed on the centrifuged urine to screen for urinary schistosomiasis within 1–2 h of collection. We will take a 24-h urine collection for urinary albumin excretion, sodium and 24-h creatinine clearance in a sample of 300 participants from Uganda during the second phase of the study.

Details of the equipment used for the measurements are outlined in Table 1 below.

### Qualitative sub-study in Uganda

The qualitative sub-study in Uganda will have two components: a study of people with abnormal renal function, and an enquiry into medicinal product supply and use sourced from traditional healers and herbalists.

This sub-study of people with abnormal renal function will include up to 50 participants found to have CKD during the recruitment of the 1000 participants for the overarching study. In order to recruit a comparator group for the 50 CKD participants, a further 50 participants without CKD of a similar age living in a neighboring house to each case will be invited to take part. Data from both cases and ‘controls’ will be collected through two in-depth interviews. These selections were made on an estimated sample to reach saturation.

For the sub-study with traditional healers/herbalists, we will approach ten traditional healers and herbalists and invite them to participate in one or more interviews to discuss the common ailments they treat and the types of herbal and other preparations that they use in their practice.

### Bio-banking

Serum, plasma and urine samples will be collected, processed and frozen and transported to the central laboratory for additional tests that cannot be conducted at the originating laboratory. All three centers have quality assurance systems for processing, sample transportation and sample storage management systems [26, 32, 33]. All sites have received ethical approval for testing of specimens for genetic studies and exploration of new markers of CKD.

### Data management

Data management will follow local established procedures ensuring quality, confidentiality and proper use of abnormal results to guide patient care when needed. Electronic data capture allows automatic data checks such as double entry of numbers and range checks. We will enter anonymized data into compatible statistical databases from centres in all three countries to for sharing between all investigators.

### Quality assurance

All three centres have quality assurance procedures that support the studies at different levels. Study staff are trained and certified according to national and international guidelines governing research. The ARK team holds regular meetings to ensure that study protocols and research standards are adhered to. We will follow standard sample storage protocols for the biorepository.

### Statistical and qualitative analysis plan

We will tabulate baseline characteristics stratified by country. We will perform initial analyses in a sample of 1500 participants selected to represent all centres, ages, sex, and eGFR categories for modification of the equation, and allow for subsequent validation using the remaining data. We plan to recombine the training and test sets for fitting a final model. We are also open to alternatives to data-splitting such as re-sampling techniques (cross-validation and bootstrapping), that would allow us to develop the model on the whole dataset and still validate predictive accuracy. Evidence from previous derivation of eGFR equations suggests that errors are multiplicative, so we will do analysis on the log (ioxehol-GFR) scale. To determine the measured GFR (iohexol-GFR); we will calculate the slope from the 3 samples at 120, 180 and 240 min points using the exact time of collection turn these into a GFR normalised to BSA using standard methods [13, 34]. We will also put in the R-value of the fit, which will be used to exclude ones where the fit is particularly poor. In our primary analysis we will evaluate the performance of the CockCroft Gault, 4v-MDRD, CKD-EPIcr, CKD-EPIcyst, CKD-EPIcr/cyst equations with and without ethnic correction factor by calculating the bias, precision and accuracy at 10% (P10) and 30% (P30) compared to iohexol GFR as done by previous studies [13]. Derivation of GFR estimating equations is rapidly developing and we will also consider evaluation against newly developed methods including the full age spectrum (FAS) and the Lund-Malmo GFR estimating equations [35, 36]. We will assess the performance of the different equations for eGFRs with and without ethnic factors using Lin’s Concordance Correlation Coefficient and Spearman correlation coefficients to evaluate the degree to which pairs of observations fall on the 45° line through the origin. We will calculate bias and relative bias. We will evaluate precision by the standard deviation of the bias (random error) and root-mean-square error. We will test the difference in P30 accuracy between eGFRs with the exact McNemar test.

We will use Bland-Altman plots to investigate the measurement error of existing equations of log(MDRD-eGFR), log(CKD-EPI-eGFR), log(CG-eGFR) and log(cystatin C-eGFR) compared to log(ioxehol-GFR). If needed, to develop modified correction factors for the setting, we will carry out regressions to predict log(ioxehol-GFR) as a function of age, gender, and creatinine/cystatin C. Predicted eGFRs will subsequently be compared with measured GFRs in the validation sample. We will investigate what happens if information on weight, height, and bioimpedance measures are added to the regression model and use computed R^2^ values to investigate whether anthropometry adds relevant information over and above existing equations.

All statistical analyses will be performed using STATA 15 SE (Stata Corp, Texas, USA).

For the qualitative study, we will record interviews with permission from the participant and transcribe and translate them. We will revise the interview guidance after the initial interviews in order to collect greater depth of data on emerging themes. We will review transcripts for accuracy and enter these into Dedoose, qualitative data analysis software. We will conduct data analysis using an iterative coding process, during the interview period. After data collection, we will perform open coding, and move to more refined codes, subthemes, and then themes. We will analyze themes and code them using a constant comparison method. We will apply the codes to interview transcripts and summary statements with representative quotes developed for each theme.

### Ethical considerations

We have obtained ethical approvals from the Uganda Virus Research Institute, Research Ethics Committee (UVRI-REC-#HS 1978) the Uganda National Council for Science and Technology (UNCST-#SS 4283), from the Malawi National Health Sciences Research Committee (#1072) and the University of Witwatersrand Human Research Ethics Committee (#M160938).

We will obtain written informed consent from participants for the collection and analysis of genetic samples as well as iohexol testing and for the use of their clinical records for research purposes. We will seek approval for all study procedures including material transfer agreements from the respective ethical review boards. In case we diagnose medical conditions through study screening, we will refer the participants to appropriate medical facilities. We will refer participants found to have advanced CKD to appropriate nephrology services as directed by the study physicians in each centre.

## Discussion

This study aims to establish the optimal approach to estimate GFR in sub-Saharan Africa. Our study has the advantage of drawing participants from three countries and both rural and urban settings within sub-Saharan Africa, which will increase the applicability of the findings across the region. Furthermore, we embedded the ARK study within established cohorts that have background data and longitudinal serial measures that we can use to characterize kidney disease over a period. The study will seek to overcome a number of the limitations of previous research.

There are challenges with measuring iohexol GFR, with the major one being accuracy of measurement of iohexol [23, 37]. Stringent measures, shared standard operating procedures across the centres, and analysis in one certified laboratory will help minimise errors.

Measurement of creatinine is another challenge. Creatinine is influenced by many factors and these can greatly influence the estimation, particularly at low eGFR [19, 37, 38]. It is particularly important that the methods used for creatinine measurements are the same across sites and are validated across the centres. We opted to use the Jaffe method for South Africa and Malawi and enzymatic method in Uganda, standardized against an isotope dilution mass spectrometry method across all sites to improve the accuracy in measurement. We will measure Cystatin C in order to assess whether the addition of cystatin C by itself or in combination with creatinine assays further refines the accuracy of estimating GFR within SSA. Several studies have shown that the addition of cystatin C to the estimation formula improves its accuracy [39, 40] and using CKD-EPI-creatinine/cystatin C improved the accuracy in a study from Congo [13]. Using a unified approach, these issues will be addressed by this study. We also plan to measure bioimpedance to define body composition and its contribution to measured GFR variation across the sites, and to assess the correlation of body composition measured by BIA with the gold standard, DXA – which will be done in South Africa [41]. Bioimpedance measurement was included in this study for measurement of lean mass and total body water and we hope to use it to optimize GFR estimation on an individual patient basis, for example in a patient with significant oedema. While this has not proven useful in high-income settings, such technologies may be useful in low-resource settings [42]. Bio-impedance will help in incorporating more parameters allowing more flexibility to work with the data to get the best formula.

Assessment of risk factors for CKD in sub-Saharan Africa has previously been hampered by inaccurate measurement of GFR. Although, not exhaustive, we included a number of risk factors for CKD in our study. We will examine the role of traditional cardiovascular risk factors including diabetes mellitus, obesity and lipid profiles along with conventional and ambulatory blood pressure monitoring. In addition we will measure the impact of infections which may be of great importance in this region where infectious diseases are common and may play a major role in disease causation alongside genetic factors [43–49].

However, there are limitations. Prolonged blood sampling for iohexol excretion may help to define GFR in patients with very poor renal function [23]. We chose not to include this in our protocol to minimize participant burden. In addition, we do not include participants from other regions of sub-Saharan Africa, including Central and West Africa but we welcome data-sharing with studies in other regions.

The African Research into Kidney disease (ARK) collaboration will provide definitive information about optimal measurement of renal function in sub-Saharan Africa, and is an opportunity for establishing close working relationships across different centres, using standardized protocols and measurements, and shared bio-repositories. We plan to build on the collaboration for this study and for future work on kidney disease in sub-Saharan Africa, and welcome additional partners from across the region including North African and Arab populations.

## Data Availability

Not applicable

## Abbreviations

ARK: African Research into Kidney Diseases
CKD: Chronic kidney disease
CKD-EPI: Chronic Kidney Disease-Epidemiology collaboration
DTPA: Technetium-99 m diethylenetriamine penta-acetic acid
EDTA: chromium-51 ethylene diamine tetra-acetic acid
EGFR: Estimated glomerular filtration rate
IDMS: Isotope dilution mass spectrometry
MDRD: Modification of Diet in Renal Disease
MGFR: Measured glomerular filtration rate
